# Perventricular Muscular Ventricular Septal Defect (VSD) Closure under Epicardial Echocardiography Guidance: A Case Report

**Published:** 2017-07

**Authors:** Akbar Molaei, Abbas Afrasiabi, Eisa Bilejani, Mahmud Samadi

**Affiliations:** *Madani Heart Center, Tabriz University of Medical Sciences, Tabriz, Iran.*

**Keywords:** *Heart septal defects, ventricular*, *Echocardiography*, *Heart defects, congenital*

## Abstract

Ventricular septal defects (VSDs) are among the most common congenital cardiac lesions. Large defects at apicomuscular regions, especially in young patients, are far from accessible to surgeons for conventional surgery. Moreover, the transcatheter closure of VSDs in these patients is difficult and carries a high risk of complications because of the large sheath size relative to the patient’s size. The periventricular approach simplifies VSD closure and, thus, eliminates the potential complications of cardiac catheterization and fluoroscopy as it is performed under echocardiographic guidance. A 3-year-old girl with a body weight of 11 kg (failure to thrive) was referred to us. She had multiple adjacent apicomuscular VSDs, the largest one being about 19 mm in diameter, and subsystemic pulmonary artery pressure (PAP). The patient underwent periventricular apicomuscular VSD closure with a Lifetech muscular VSD occluder (size 22 mm) under epicardial echocardiography guidance without cardiopulmonary bypass. Post procedure, the PAP was decreased to mild level. The residual shunt was mild across the adjacent small defects. She was discharged after 7 days without complications. At 2 years’ follow-up, the patient was hemodynamically stable and had a normal PAP (PAP = about 16 mmHg) by transthoracic echocardiographic assessment.

## Introduction

Numerous hybrid procedures have been introduced in recent years for the treatment of cardiovascular lesions and have proved especially essential to the management of congenital heart diseases (CHDs).^1^ Ventricular septal defects (VSDs) are the most common CHD and are deemed suitable lesions for hybrid surgery, not least the muscular types.^2^

Large defects at apicomuscular regions, particularly in young patients, are difficult to access by surgeons through conventional surgeries.^3^ The percutaneous closure of VSDs in these patients is difficult and carries a high risk of complications because of the large size of the sheath relative to the size of the patient.^4^ In the meantime, catheters are difficult to manipulate, which in turn increases cardiac catheterization time, radiation, and risk of dysrhythmia.^5^ Not only does the periventricular approach simplify VSD closure but also it eliminates the potential complications of cardiac catheterization and fluoroscopy as it is performed under echocardiographic guidance. The periventricular technique was introduced in 1997, and the first patient to undergo the intraoperative device closure of a muscular VSD without cardiopulmonary bypass (CPB) was reported by Amin and colleagues in 1998.^1^ This procedure can be performed under echocardiography guidance through either transesophageal echocardiography (TEE) or the epicardial approach.^6^

Herein, we report the first periventricular muscular VSD closure under epicardial echocardiography guidance in Iran.

## Case Report

**Figure 1:**
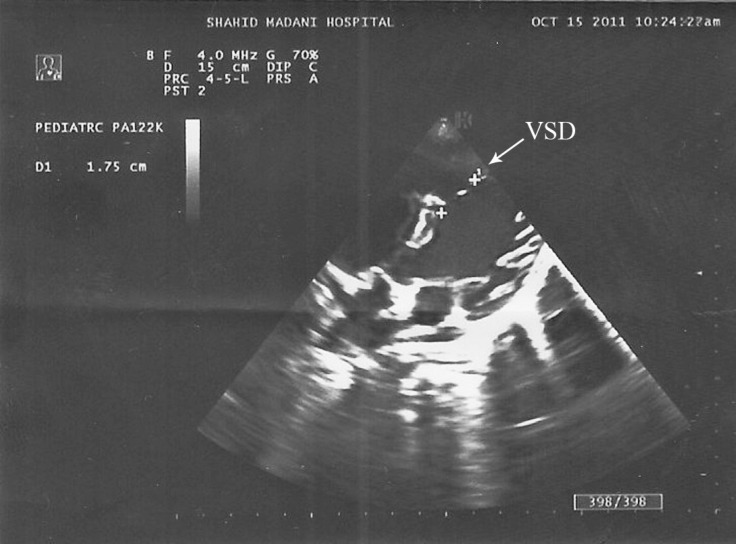
Parasternal short-axis view of the heart by epicardial echocardiography shows the large apicomuscular VSD.

**Figure 2:**
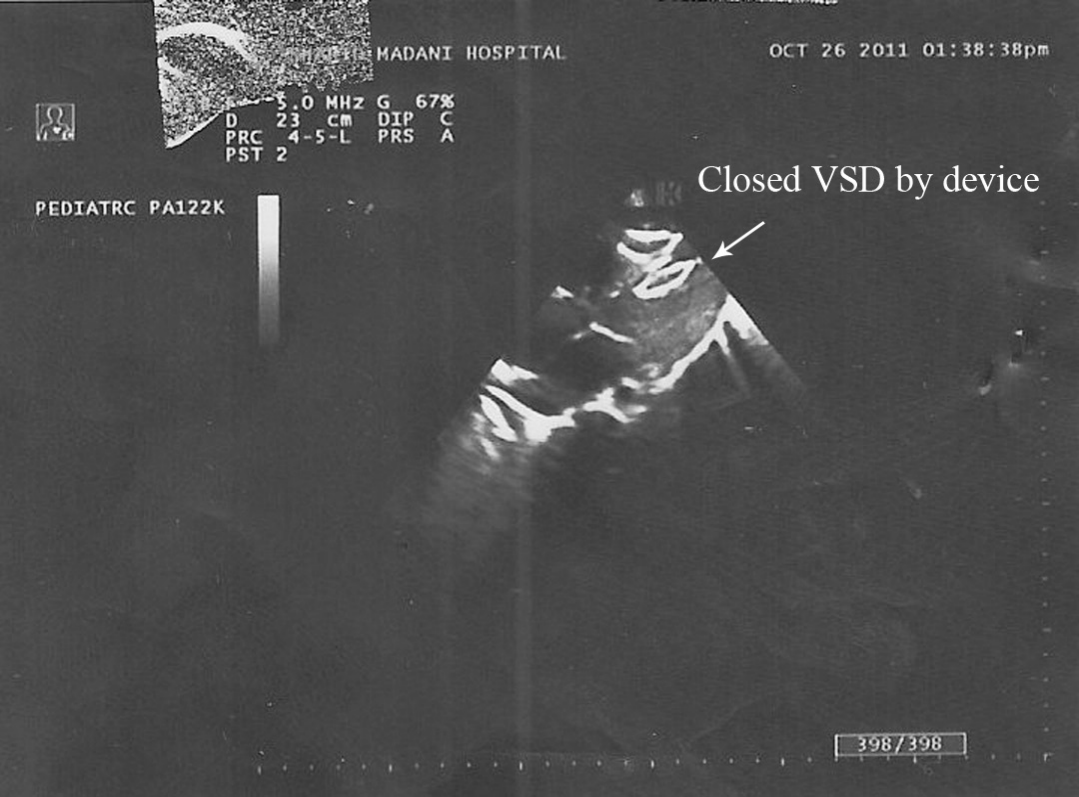
Apical 4-chamber view by epicardial echocardiography shows the device at the position of apicomuscular defect after defect closure via the periventricular procedure.

A 3-year-old girl with a body weight of 11 kg was referred to us. She had multiple adjacent apicomuscular VSDs, the largest one being approximately 19 mm in diameter (Figure 1). Her pulmonary artery pressure (PAP) was subsystemic and due to the location of the defect and the age and size of the patient, neither conventional surgery nor percutaneous transcatheter procedure was suitable. The patient underwent periventricular apicomuscular VSD closure with a Lifetech muscular VSD occluder (size = 22 mm) under epicardial echocardiography guidance (SonoSite echocardiography machine) without CPB (Figure 2). After the closure of the defect, the PAP was decreased significantly. The residual shunt was mild across the adjacent small defects.

VSD, Ventricular septal defect

VSD, Ventricular septal defect

The patient underwent generalized anesthesia and endotracheal intubation. Subsequently, mid sternotomy was performed by a cardiac surgeon. The defect size was reevaluated using epicardial echocardiography, and the point of sheath insertion was detected. A 5–0 polypropylene purse-string was placed at the chosen location by the surgeon. An 18-gauge needle (Cook, Bloomington, IN, USA) was introduced into the right ventricular cavity, and a 0.035-inch angled glide wire (Boston Scientific, Medi-Tech, Natick, MA, USA) was passed through the needle and manipulated into the left ventricular (LV) cavity through the defect. A 12-F short (8 cm) introducer sheath with a dilator was passed over the wire and carefully advanced into the LV cavity. Care was taken to keep the tip of the dilator in the middle of the LV cavity (monitored by epicardial echocardiography) because it was likely to perforate the LV free wall. The dilator was removed and the sheath tip positioned in the LV cavity. The appropriate device (Lifetech muscular VSD occluder size = 22 mm) was selected. The device was then screwed to the cable and pulled inside a 12-F loader under blood seal to prevent any air bubbles. The device was advanced inside the short delivery sheath until it was seen by echo to be close to the tip of the delivery sheath. The LV disc was deployed in the mid-LV cavity by gentle retraction of the sheath over the cable. The entire assembly (cable/sheath) was withdrawn gently until the LV disc was against the septum. By further retraction of the sheath over the cable, the waist was deployed inside the septum. Continuous epicardial echocardiography was of vital importance to confirm the device position. Once the position was confirmed, by further retraction of the sheath, the right ventricular disc was deployed. After the confirmation of the device position via epicardial echocardiography, the device was released by counterclockwise rotation of the cable using the pin vise. A complete epicardial echocardiographic study was performed in multiple planes to confirm device placement and probe for residual shunting or any obstruction or regurgitation caused by the device.

Following the release of the device and the confirmation of the device stability, the sheath and the cable were extracted from the heart and the purse-string was tightened by the surgeon to achieve complete homeostasis. Later, the chest was repaired anatomically and the patient was transferred to the pediatric intensive care unit.

**Figure 3:**
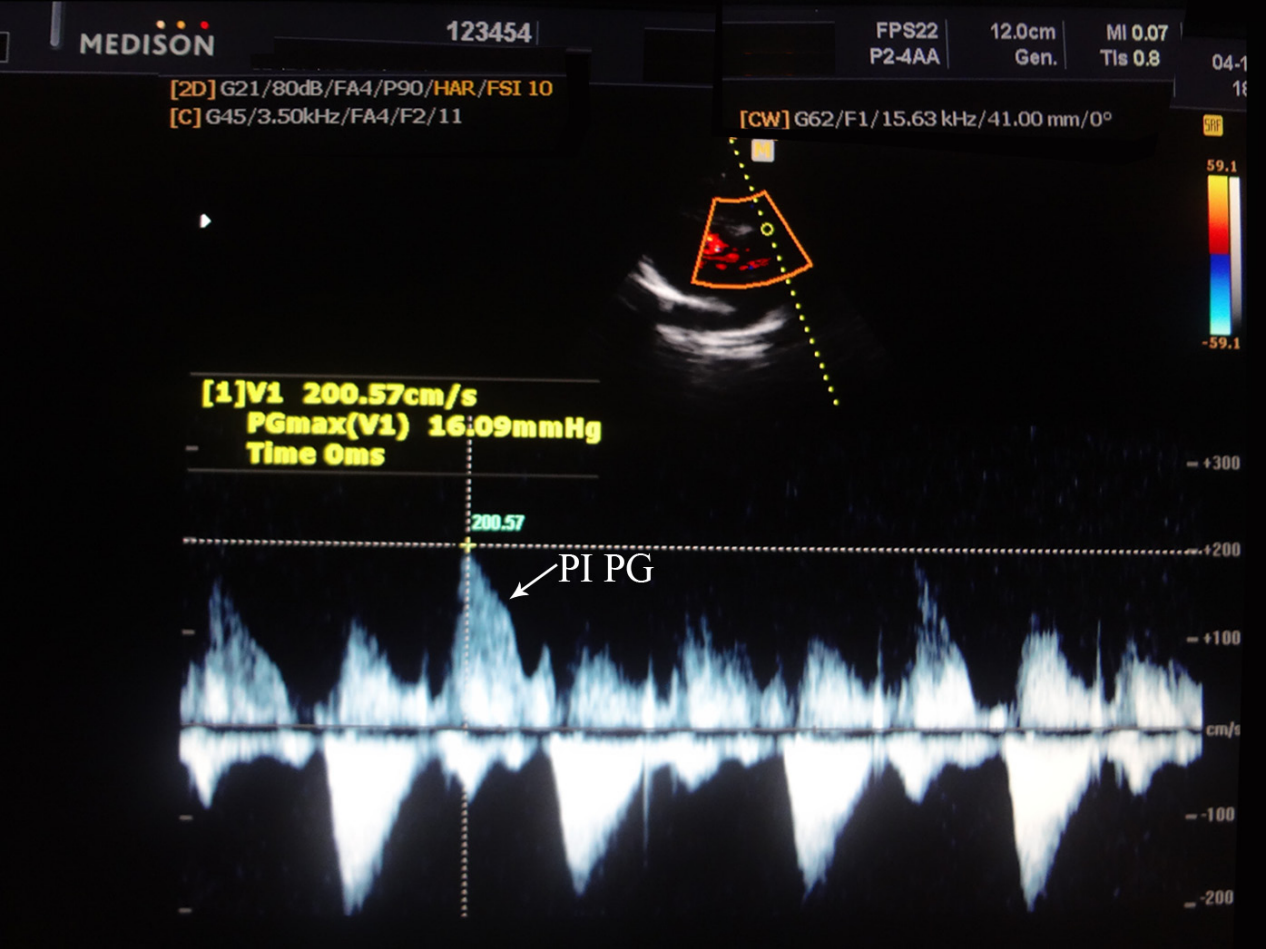
Continuous wave Doppler tracing of the pulmonary valve of the patient by transthoracic echocardiography at 2 years’ follow up shows mild pulmonary valve insufficiency with a 16 mmHg pressure gradient.

At 2 years’ follow-up, the patient was hemodynamically stable and she had a normal PAP (PAP = about 16 mm Hg) in transthoracic echocardiographic assessment (Figure 3).

PIPG, Pulmonary valve insufficiency pressure gradient

## Discussion

VSDs are the most common CHD. There are some therapeutic options for these defects. Conventional surgical patch closure needs CPB and has limitations for apicomuscular VSDs, specifically in infants and young children.^1^ Also, percutaneous transcatheter device closure is not suitable for infants and young children due to unsuitable vascular access.^2^

A relatively new method, periventricular VSD closure by device has removed these limitations. This method was first described by Amin et al.^1^ in animal models. Since then, this procedure has been performed in different centers with different devices via sternotomy or subxiphoid approaches without CPB.^3^

This procedure can be performed under TEE or epicardial echocardiography guidance. TEE confers an acceptable assessment of the size and location of the defect and the adjacent structures, and it obviates the need for procedure interruption. However, unfortunately there are limitations to the application of the TEE probe, especially in pediatrics.^6^^-^^7^ In contrast to TEE, epicardial echocardiography is noninvasive and does not have probe limitations. Nonetheless, the intermittent interruptions of the procedure are inevitable. Further, epicardial echocardiography gives rise to hypotension and bradycardia due to direct right ventricular compression.^8^^-^^10^ This procedure has potential complications such as LV posterior wall perforation, injury to the tricuspid or mitral valve apparatuses, conduction disturbances, infection, and device dislodgement or embolization.^2^

To the best of our knowledge, our experience reported herein is the first of its kind in Iran. We assume that by using TEE and fluoroscopy (in the hybrid room), the procedure can be performed not only more easily but also much safer and faster.

## Conclusion

Periventricular VSD closure by device under TEE or epicardial echocardiography guidance via small sternotomy incisions is a feasible and safe approach in selected patients.

## Notes:

***This paper should be cited as: ***Molaei A, Afrasiabi A, Bilejani E, Samadi M. Perventricular Muscular Ventricular Septal Defect (VSD) Closure under Epicardial Echocardiography Guidance: A Case Report. J Teh Univ Heart Ctr 2017;12(3):138-141.
